# Sampling strategy of an epidemiological survey using a satellite image program

**DOI:** 10.11606/S1518-8787.2019053000834

**Published:** 2019-05-02

**Authors:** Ticiane de Góes Mário Ferreira, José Mariano da Rocha, Silvia Cardoso de David, Jociana Boligon, Maísa Casarin, Alessandra Pascotini Grellmann, Janice Marin, Thiago Machado Ardenghi, Fabricio Batistin Zanatta, Carlos Heitor Cunha Moreira

**Affiliations:** I Universidade Federal de Santa Maria . Curso de Odontologia. Programa de Pós-Graduação em Ciências Odontológicas . Santa Maria , RS , Brasil; II Universidade Federal do Rio Grande do Sul . Faculdade de Odontologia . Departamento de Periodontia . Porto Alegre , RS , Brasil; III Universidade Federal de Pelotas . Faculdade de Odontologia . Departamento de Semiologia e Clínica . Pelotas , RS , Brasil; IV Universidade Federal de Santa Maria . Curso de Odontologia. Departamento de Estomatologia . Santa Maria , RS , Brasil

**Keywords:** Sampling Studies, Satellite Applications, Dental Health Surveys, Rural Health

## Abstract

**OBJECTIVE:**

To describe the sampling strategy of an epidemiological survey with the aid of satellite images, including details of the multistage probability sampling process.

**METHODS:**

A probability sample of individuals living in the rural area of Rosário do Sul, state of Rio Grande do Sul, Brazil, aged 15 years old or more, was evaluated. Participants answered questionnaires (medical history, sociodemographic characteristics, habits, alcohol use, quality of life, stress, rumination, and self-perceived periodontal diseases), and were subjected to clinical oral examinations as well as anthropometric measurements (blood pressure, height, weight, abdominal and waist circumferences). Oral evaluation comprehended a complete periodontal exam at six sites per tooth, including the following assessments: furcation involvement; dental abrasion; tooth decay, including the indexing of missing and filled surfaces; O’Brien index; gingival abrasion; oral cavity and lip lesions; complete periapical radiographic exam, and use of prostheses. Besides this oral clinical approach, subgingival plaque, crevicular gingival fluid, saliva, and blood samples were collected. Examiners were trained and calibrated during previous evaluations. A pilot study allowed the logistic of the performed exams to be adjusted as needed.

**RESULTS:**

Among 1,087 eligible individuals, 688 were examined (63.3%). Age, sex, and skin color data were compared to data from the last demographic census (2010) of the Brazilian Institute of Geography and Statistics, which served to validate the sampling strategy.

**CONCLUSIONS:**

The careful methods used in this study, in which satellite images were used in the delimitation of epidemiological areas, ensure the quality of the estimates obtained and allow for these estimates to be used in oral health surveillance and health policies improvements.

## INTRODUCTION

Oral disorders remain highly prevalent, affecting 3.5 billion people worldwide ^1^ . Almost 50% of the world’s population suffers from disabilities related to oral conditions ^2^ . Untreated caries in permanent teeth ^2^ are the most prevalent oral illness, and went through an increase of 14.5% between 2005 and 2015 ^1^ . In the same period, periodontal disease, edentulism or severe tooth loss, and lip or oral cavity cancer had an increase of 25.4%, 27.3%, and 38.6%, respectively ^1^ . The numbers are more disturbing when the years of healthy life lost due to premature disability (disability-adjusted life years, DALY) are considered. The main cause of DALY related to oral conditions is tooth loss (7.6 million DALY), followed by severe chronic periodontitis (3.5 million DALY) and untreated caries (1.7 million DALY) ^2^ .

Oral health problems affect health-related quality of life ^3^ and are considered a public health issue. In this sense, they must be a priority of the universal health coverage debate, as well as a focus of policy development ^4^ . The formulation of policies and oral health goals might be more effective if based on good-quality descriptive epidemiology ^2 , 5^ . However, researches with probabilistic population-based samples are lacking in some regions, particularly rural areas in Latin America ^6^ . Sampling approaches for evaluating the population living in these regions can differ from the ones used in urban areas, due to the absence of precise territorial divisions, such as squares, neighborhoods, and dwelling numbers. In these cases, satellite images can be useful, since they allow for wide, cost-effective access to different remote areas.

Methodological studies describing these issues in the countryside are severely lacking. The investigation of health conditions of rural inhabitants is necessary especially due to these populations’ difficult access to medical and oral care ^7^ , considering that the improvement of oral conditions is strongly associated with better access to health care systems ^8^ . Additionally, more research is needed to evaluate the impact of oral diseases and to explore the determinants of oral health inequalities ^4^ .

Considering the importance of methodological quality for oral health estimates and the lack of a detailed description of strategies for sampling rural populations, this paper aimed to describe the sampling procedures used in an epidemiological survey of a rural population from Southern Brazil.

## METHODS

### Study Design and Survey Population

This was a population-based cross-sectional survey. The surveyed population was comprised of individuals aged 15 years or older, living in a rural area of Rosário do Sul. Rosário do Sul is a city located at the west border (geographic coordinates 30° 15’ 28” S, 54° 54’ 50” W) of the Brazilian state of Rio Grande do Sul, in Brazil’s South, neighboring Argentina and Uruguay. The city has approximately 4.4 thousand km ^2^ and 40,000 inhabitants, among whom 4,776 live in rural areas ^9^ . This is a difficultly accessed population scattered over a wide territorial extension, considering that the rural demographic density of Rosário do Sul is around one inhabitant per kilometer square [unofficial data provided by the Santana do Livramento office of the Brazilian Institute of Geography and Statistics (IBGE), responsible for the Rosário do Sul census]. The majority of its populated rural areas are distant from the urban center, while access roads are usually in precarious conditions. These characteristics can hamper transport to the city, restricting inhabitants’ use of crucial urban services, such as health care.

The IBGE Population Census operation is performed according to the administrative divisions defined by municipal law (i.e., districts, urban and rural areas), which are further subdivided into smaller areas, called census enumeration areas (CEA). Each CEA is a continuous area with borders that are easily identifiable during field operation, and have dimensions and numbers of households (nH) that make it possible for census interviewers to carry out their activities over a feasible timespan, respecting the operation’s overall schedule ^10^ .

Rosário do Sul has six districts and 36 rural CEA (RCEA), which vary in area (0.0831–338.034 km ^2^ ) and nH [median of 46 (1–189)]. The area’s nH differs considerably from what is usually observed in IBGE RCEA sudvisions, which commonly have 150 to 250 dwellings.

### Sampling Strategies

The sample of ≥ 15-years-old dwellers from Rosário do Sul’s rural area was obtained using a multistage probability sampling method, as well as the territorial maps (.kmz format, Google Earth program) provided by IBGE ^11^ . The municipal government of Rosário do Sul provided additional information for the sampling process.

**Figure 1:**
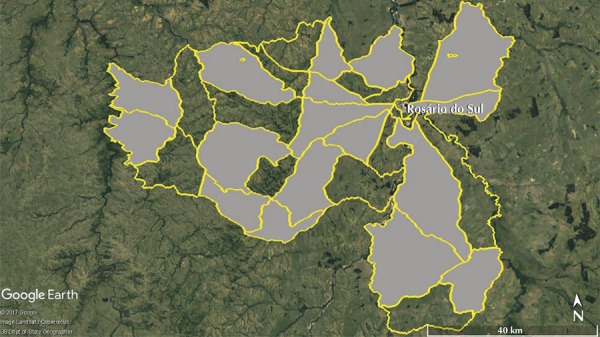
The 30 eligible and the 17 randomized (gray color) rural census sectors. The latter appear outlined in yellow.

In order to protect census informers, the IBGE was unable to provide data on individuals from six of the RCEA, which had less than five permanent dwellings ^12^ . Thus, these territorial units were not included in the study’s sample. The remaining 30 RCEA were divided into three strata (small, medium, and large), according to nH tertiles. Three randomized sequences were generated in Research Randomizer ^a^ , in order to select 17 RCEA [56.7% (three small, seven medium, and seven large), Figure 1 ]. This enabled the inclusion of all six of the municipality’s districts. In this way, the sampling process accounted for the number of individuals and dwellings registered in each RCEA, according to IBGE data.

The OpenEpi program ^b^ was used to calculate sample size, considering a rural population aged ≥ 15 years, amounting to approximately 4,000 inhabitants ^9^ , and periodontal disease (50% prevalence) as the worst case scenario for the main outcome. A 4% precision level was used, together with a 1.3 design effect for the 95% confidence interval. The sample size calculation was adjusted for finite populations using a standard formula [n adjusted = n/1+(n/N); in which *n* is the calculated sample size, and *N* is the population size]. This resulted in an estimated sample size of 580 individuals. The sample was then increased by 15%, to 667 individuals, so as to account for non-response.

The total nH in each RCEA stratum was obtained from IBGE data. The estimated nH to be visited in each of the 17 randomized RCEA was proportional to sample size and population density. Thus, the nH in each RCEA was estimated considering the mean number of ≥ 15 years old individuals living in each dwelling (n = 3).

Seven randomized RCEA (41.18% of evaluated RCEA) had community health workers who provided us with local lists, allowing for the random selection ^a^ of eligible dwellings (i.e., dwellings with at least one eligible dweller). In the other RCEA, the most densely populated region was determined according to house clusters viewed on the satellite images provided by IBGE (.kmz format, Google Earth program), which contained pre-delimited RCEA ^11^ . This region was then confirmed *in loco* by researchers as the correct starting point for the selection of dwellings. First, dwellings on the right side of the street were visited and, if necessary, the team went back to the starting point in order to visit households on the left side. After establishing the direction, all dwellings at the right and left sides of the road were considered eligible. All dwellings within the RCEA area with at least one dweller from the right and left were accounted for consecutively, in a straight line, until the pre-specified nH or subject number were reached. When these strategies failed to obtain the nH necessary to compose the sample, secondary roads within a radius of five kilometers at the right or left of the main road were also accessed.

All individuals ≥ 15 years living in eligible dwellings were considered eligible to the study. Exclusion criteria were the following: presence of a systemic disease or condition disavowing clinical examination or requiring a prophylactic regimen of antibiotics in preparation for it; diagnosis or family report of psychiatric or mental problems, and alcohol or drug intoxication.

#### Non-response data

A questionnaire pertaining sex, age, schooling, skin color, family income, tobacco use, and the number of teeth present in the mouth was applied to non-responders.

## Data Analysis

Descriptive analysis was done by means of absolute numbers and frequency distributions, adjusted for the complex sample. Sample weights were the inverse of the probability of selection, considering the RCEA size. Thus, the weight was calculated by dividing the RCEA’s population size by the number of individuals sampled in each RCEA. The non-response rate in each RCEA was considered in sample weight calculation (weight = 1 / sample fraction × non-response rate). Data for skin color, sex, and age were compared to data from the 2010 IBGE census, available from its institutional website ^13^ . These comparisons were performed via chi-square test, adjusted for the complex sample. All analysis employed IBM ^®^ SPSS ^®^ Statistics software, version 21.

## Operational Procedures

The data were collected by six dentists between March 2015 and May 2016. Researchers were divided according to their assigned functions: interviewers (TGMF and SCD), clinical examiners (MC and JB), physical examiner (APG), and dental radiographer (JM, CASB, CFW, and FBC). The latter function was assumed by four examiners, who took turns, so that no one would be exposed to radiation for more than one day’s work.

Clinical and radiographic examinations were performed in a mobile unit, consisting of a trailer equipped with a complete dental unit (dental chair, artificial light, compressor, dental x-ray machine, and other basic amenities). The mobile unit was moved to a central point in each RCEA, following the survey schedule. A team of dentists previously visited the households, in order to explain the aims of the study and invite subjects to participate. Individuals who agreed to take part in the study were assigned an examination appointment. In the first visit, dentists collected telephone numbers and numbers of residents in each dwelling. The contact information of a neighbor to be contacted when there was nobody at home was requested. Individuals who failed to attend the examination appointment or were not at home during the first attempt received new home visits or calls to encourage participation. Dwellings with no one present during the first visit received a minimum of three additional visitation attempts, as well as five telephone calls. In RCEA with community health workers, the latter were responsible for scheduling the examinations, according to the list of randomly selected dwellings. In these cases, the aims of the study were explained during the examination appointment itself.

## Interviews

**Figure 2:**
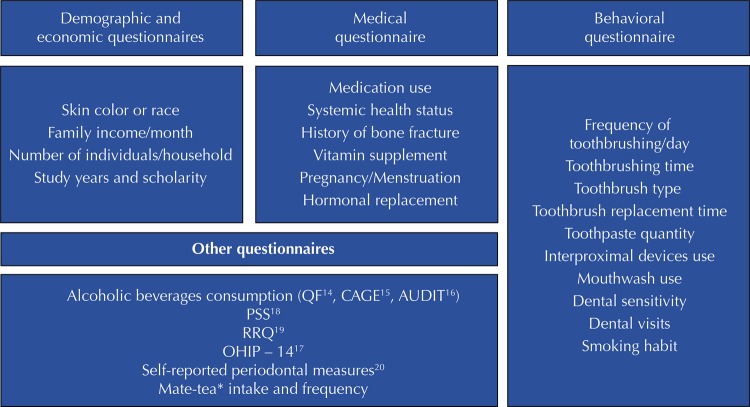
Questionnaires scheme.

QF: Quantity Frequency; CAGE: Cut-down, Annoyed, Guilty, Eyeopener; AUDIT: Alcohol Use Disorders Identification Test; PSS: Perceived Stress Scale; RRQ: Rumination – Reflection Questionnaire; OHIP: Oral Health Impact Profile

Trained dentists conducted face-to-face interviews individually, in order to apply structured questionnaires. The interviews included data on sociodemographic, economic, medical and behavioral factors, as well as alcohol intake ^14^ , oral health impact on quality of life (OHIP-14 ^17^ ), stress (PSS ^18^ , RRQ ^19^ ), and periodontal disease perception ^20^ ( Figure 2 ).

* Tipical beverage in the Brazilian state of Rio Grande do Sul, also known as *chimarrão* .

## Evaluations

Clinical examination comprehended previous use of prostheses, evaluation of oral cavity soft tissues, as well as lip lesions (description of localization and probable clinical diagnosis), tooth count (are all teeth present in the mouth, including third molars), periodontal exam, dental abrasion, dental caries [index of decayed, missing and filled surfaces (DMFS) ^21^ ], dental trauma (O’Brien index ^22^ ), gingival abrasion (Danser et al. ^23^ method), and periapical radiograps.

The complete periodontal exam included: visible plaque index ^24^ (VPI), retentive plaque factors (presence of supragingival calculus, caries, cavitated lesions, restoration with excess or lack of restorative material, and restorable residual roots), gingival bleeding index ^24^ (GBI), pocket probing depth (PPD), bleeding (BoP), and suppuration on probing (dichotomously, after PPD measurement), clinical attachment loss (CAL, defined as the distance from the cementoenamel junction to the bottom of the pocket), and furcation involvement ^25^ . Gingival recession was calculated by subtracting the CAL from the PPD during data analysis. All these periodontal parameters were evaluated at six sites per tooth, excluding third molars, with a UNC-15 probe (Neumar ^®^ , São Paulo, Brazil) and dental mirror. In edentulous individuals, only evaluation of oral cavity, lip lesions, and gingival abrasion were performed.

Additionally, biological samples (blood, gingival crevicular fluid, subgingival plaque, and unstimulated saliva), and anthropometric measurements (blood pressure, height, weight, abdominal and waist circumferences) were collected, and the body mass index was calculated.

## Training and Calibration

The examiners received training prior to conducting the evaluations. The training comprehended definitions of clinical and physical parameters, measuring instruments, correct measuring techniques, clinical photographs, and questionnaire application. The team also received a manual containing instructions about data collection and instrument management for use in fieldwork. Firstly, there were four one-hour sessions dedicated to theoretical training. Each session was used to discuss the indexes and variables that would be evaluated, and to define diagnostic criteria. In the second stage, the team participated in practical and clinical activities. The examiners practiced the physical measurements (anthropometric and saliva collection) as well as questionnaire application among themselves. A radiology professor trained the examiners responsible for executing radiographic exams in the use of the x-ray machine’s digital sensor, positioner, and software, performing the complete periapical radiographs of three individuals. The practical training for gingival abrasion and O’Brien index was performed using clinical photographs. Training for VPI, GBI, BoP/suppuration and furcation degrees was done in five individuals, with the presence of an experienced examiner.

Calibration for PPD and CAL was performed before data collection procedures, and also during the study. Intra- and inter-examiners reproducibility was tested from repeated measurements, with a minimal interval of one hour, using ≥ 1,000 sites, in approximately seven individuals. In the pre-data collection calibration, one experienced examiner, who gave 14 individuals full-mouth examinations, was considered the gold standard (TGMF). Each additional examiner (MC and JB) evaluated the same subjects’ two crossovered quadrants (n = 14) to obtain the minimal number of sites necessary. Calibration performed during the study involved only two examiners (MC and JB), who collected the study’s clinical data. DMFS calibration was performed prior to fieldwork, in twenty extracted teeth (five surfaces/tooth). The second DMFS evaluation was performed after a two-day interval, with BE being considered the gold standard examiner for inter-examiners reproducibility. The intra- and inter-examiners agreements for PPD and CAL were verified via the intra-class correlation coefficient (ICC). For DMFS, the Kappa coefficient was used. A reproducibility of ICC/Kappa > 0.80 was considered satisfactory.

## Pilot Study

Besides the training and calibration process, the research team also participated in a pilot study’s data collection. This pilot was carried out in one day, in a RCEA outside the randomized area. The researchers decided the starting location for data collection, parked the mobile unit, and invited participants from the nearby area. The evaluations were performed on 15 individuals. This experience was crucial for deciding the functions of each group member, and also for finding the most effective sequence of evaluations.

## Ethical Considerations

The study was conducted in accordance with the Declaration of Helsinki (1964, revised in 1975, 1983, 1989, 1996, and 2000) and approved by the Ethics Committee in Research of the Universidade Federal de Santa Maria. Participants signed an informed consent form (ICF). Individuals < 18 years old needed the authorization of the person responsible for them, via the signature of a specific ICF. All participants received a written report detailing their oral status and were referred to treatment if any health alteration was identified.

## RESULTS

ICC values for intra-examiner reproducibility varied from 0.89 to 0.93 (PPD), and 0.88 to 0.99 (CAL). Inter-examiner ICC values varied from 0.89 to 0.96 (PPD) and 0.84 to 0.97 (CAL). The Kappa coefficient for DMFS (intra- and inter-examiners) varied between 0.81 and 0.88.

Among nearly 4,000 individuals ≥ 15 years old living in the rural area, 1087 met the eligibility criteria, and 399 did not participate of the study. The principal reason for non-participation was refusal (62.4%); other reasons were impossibility to go to the exam unit (16.8%), non-specified reasons (15.5%), absence after several contact attempts (4.8%), and having only completed the answering of questionnaires step (0.5%). Thus, 688 (63.3%) individuals were clinically examined. Among non-responders, 66 (16.5%) refused to answer the specific questionnaire, while 40 individuals (10.0%) failed to provide all the required information (at least one unanswered question).

Table 1 shows the demographic characteristics of the study’s sample, eligible individuals, and non-responders, as well as IBGE data for eligible RCEA. Most individuals (among examined, eligible, and IBGE-evaluated) reported having white skin color. Fifty percent of non-responders were white, while 20.6% did not provide skin color information. In the IBGE and eligible individuals data, there was a slight predominance of men. In the study’s sample, examined individuals were equally divided between males and females. Among non-responders, however, more than 60% were men. According to the IBGE, eligible individuals, and examined individuals data, approximately 70% had between 25 and 64 years of age. Comparisons between the sample and non-responders showed no statistical difference in characteristics other than sex.

**Table 1:** Demographic characteristics (study’s sample, eligible individuals, non-responders [NR]), and IBGE data).

Variable	Sample	NR	p*	Eligible	Valid	IBGE
				
	n (%)	n (%)		n (%)	%	n (%)
Skin color			0.30			
White	473 (67.5)	200 (50.1)		673 (61.9)	67.0	3,032 (82.8)
Non-white	215 (32.5)	117 (29.3)		332 (30.5)	33.0	632 (17.2)
Skin color not reported	0 (0)	82 (20.6)		82 (7.5)	-	0 (0)
Gender			< 0.01			
Male	339 (49.6)	244 (61.2)		583 (53.6)	-	2,070 (56.5)
Female	349 (50.4)	155 (38.8)		504 (46.4)	-	1,594 (43.5)
Age (years)			0.19			
≤ 24	67 (9.6)	38 (9.5)		105 (9.7)	10.3	561 (15.3)
25–34	94 (13.0)	42 (10.5)		136 (12.5)	13.3	561 (15.3)
35–44	115 (16.7)	59 (14.8)		174 (16.0)	17.0	684 (18.7)
45–54	154 (22.5)	61 (15.3)		215 (19.8)	21.1	691 (18.9)
55–64	133 (20.0)	50 (12.5)		183 (16.8)	17.9	575 (15.7)
65–74	84 (12.4)	50 (12.5)		134 (12.3)	13.1	392 (10.7)
≥ 75	41 (5.7)	33 (8.3)		74 (6.8)	7.2	200 (5.5)
Age not reported	0 (0)	66 (16.5)		66 (6.1)	-	0 (0)

Total	688	399		1,087		3,664

IBGE: Brazilian Institute of Geography and Statistics (2010) ^13^ .

Valid %: excluding unreported data.

* Chi-square test between sample and NR.

## DISCUSSION

This paper discussed methodological concepts of an epidemiological survey emphasizing oral health in a probability sample of a large rural area of Brazil’s southern region. The latest statistical data ^2^ on global oral health shows the significant burden of untreated dental caries, severe periodontitis, and edentulism. These oral conditions affect 3.5 billion people worldwide, and pose a complex public health challenge to policy makers, indicating the need for greater efforts and different approaches if this scenario is to be improved by 2020 ^2^ . Accurate epidemiological surveys such as the one performed in this study provide reliable support for assessing the current oral health status of a population, as well as its future healthcare needs ^5 , 21^ . In this sense, this research can be seen as an example among the approaches necessary for the improvement of oral health, since it allows for the identification of healthy and ill individuals in a population-based sample. Data on oral health status are important for the surveillance of disease patterns ^21^ , and therefore essential for the definition, implementation, and evaluation of public health actions, which can be direct towards both collectives and individuals, and involve preventive measures or direct care ^21 , 26^ .

The careful methods employed here – including well-thought-out, multistage sampling strategies, consideration of the complex sample in the data analysis, training and calibration of examiners, and full-mouth periodontal exams – ensure the quality of the generated estimates and data, making this study a useful health surveillance tool. It is well known that properly designed surveillance studies can assist governments, health authorities, and health professionals in formulating policies and programs for disease prevention. Additionally, these studies contribute to measuring the efficacy of efforts to control prevalent illness and restore the quality of life ^21^ .

The sampling strategy used in the study was considered the most adequate to Rosário do Sul, given its huge territorial extension (it is the seventh largest area of the state of Rio Grande do Sul ^27^ ), low rural demographic density, and its population’s compromised access to urban areas and healthcare. The strategy used to direct and order the sample allocation could be seen as a limitation of this study; however, the same methodology has been applied successfully by IBGE census interviewers in the assessment of RCEA ^10^ . RCEA identification needs to account for natural landscapes and imaginary lines that differ from official urban area divisions (squares, neighborhoods and household identification numbers). The Google Earth program was useful for circumventing the difficulties of this sampling strategy, as its images ensure the non-null probability of dwelling selection. The satellite images (maps) from the program enabled the identification of eligible dwellings and RCEA delimitations and, consequently, the sampling framework. Other epidemiology studies have also employed Google Earth images, or similar approaches, in order to construct the sampling framework ^28^ , especially in situations of limited availability of populational data, as in this study. These new technological tools have been considered useful to investigate infectious disease epidemiology ^32^ , and also for health surveillance ^33^ . The satellite image approach used here has several strengths: it facilitates sampling area delimitation, and provides detailed, high resolution, up-to-date images that are free to use and belong to the public domain. However, there are some disadvantages, such as the considerable time spent in the manual identification of dwellings, as well as the issue of possible changes in dwellings’ actual conditions taking place in the interval between image acquisition and the moment of sampling.

Comparisons between data from the study and from IBGE aimed to validate the strategy used for obtaining the rural area’s probability sample. The compared characteristics appeared in very similar frequencies across the examined sample, eligible individuals, and IBGE data. There was predominance of the white skin color in the three subgroups mentioned above. The slightly higher proportion of the white skin color seen in the IBGE data could be explained partially by the quantity of non-responders, i.e., individuals who failed to report information regarding this variable. This study employed the same skin color choices used in the IBGE census; however, several individuals demonstrated having doubts about this question. In these cases, the interviewers explained each option until the participant completely understood them, and waited for their answer, without considering the interviewer concepts of skin color. We are not sure whether IBGE census interviewers use the same approach, but this could also explain the frequency of white skin color found in our sample.

According to IBGE data, there are slightly more men than women living in the studied rural area; however, the examined sample showed was equally divided among both sexes, which could be justified by a greater quantity of men among non-responders. As Rosário do Sul is driven almost entirely by agriculture, and men are more involved in agricultural activities ^34^ , it is possible that a significant portion of men were unable to participate in the study due to farm work demands.

In regards to age, the slight difference between the IBGE sample and our sample, in regards to eligible individuals belonging to the first age bracket (less than or equal to 24 years of age), could be due to the combination of the ongoing rural exodus and census data lag. The young population in Brazilian rural areas is declining, especially among individuals aged ≤ 25 years ^35^ .

This study’s data collection process started five years after the last IBGE census (2010), which was used for the comparisons, making population-estimate lag a feasible problem. We were unable to update the dwelling registry of the randomized RCEA sample, which could be considered a further limitation. Nevertheless, there are no official data showing a change in population between the census period and the study period, and in fact the municipal government uses the last available census for its administrative planning. Thus, we believe this limitation had no effect on the external validity of the study, especially since there were no differences between the selected subjects and the demographic characteristics of the reference population. Moreover, possible variations among RCEA were adjusted considering design effect and weight for complex samples.

The two clinical examiners received the minimum reliability score (0.80) previously established in the calibration of intra- and inter-raters for PPD, CAL, and DMFS. Reproducibility values were excellent, considering the cut-off points established in the literature. Kappa values between 0.81 and 1.00 represent an almost perfect agreement strength ^36^ ; an ICC between 0.75 and 0.90 indicates good reliability, while values greater than 0.90 depict excellent reliability ^37^ .

Due to its multidisciplinary character, this survey allowed for a complete health investigation, and therefore a health status report was delivered to each participant. Copies of these reports will also be delivered to the municipal health service department, accompanied by suggestions and strategies for the management of the main health problems observed. The summary of study results will be available to the general public through a local radio program, which reaches both the urban and the rural area. Apart from the scientific papers resulting from this study, we believe that these procedures may contribute to the development of local health care programs.

## Footnotes

a Urbaniak GC, Plous S. Research Randomizer (Version 4.0) [Computer software]. Lancaster, Pennsylvania. 2014. Available from: http://www.randomizer.org/

b Dean AG, Sullivan KM, Soe MM. OpenEpi: Open Source Epidemiologic Statistics for Public Health. Version 3.01. [updated 2013 Apr 06; cited 2014 Aug 8]. Available from: http://www.openepi.com/Menu/OE_Menu.htm
